# Iron Restriction Alleviates Atherosclerosis in ApoE KO Mice: An iTRAQ Proteomic Analysis

**DOI:** 10.3390/ijms232415915

**Published:** 2022-12-14

**Authors:** Gang Luo, Lu Xiang, Lin Xiao

**Affiliations:** Xiangya School of Public Health, Central South University, Changsha 410078, China

**Keywords:** atherosclerosis, iron, iTRAQ, Gal-3, VCAM1

## Abstract

The “iron hypothesis” of atherosclerosis has long been controversial. Several studies have shown that dietary iron restriction or low-iron diets can effectively alleviate atherosclerosis in rabbits and mice. However, the underlying molecular mechanisms of these phenomena remain to be elucidated. In this study, we further evaluated possible correlations between a low-iron diet and atherosclerosis alleviation by using a quantitative proteomic approach. For this purpose, apolipoprotein E knockout (ApoE KO) mice were divided into three groups and fed a normal diet (ND), a high-fat diet (HFD), or a high-fat +low-iron diet (HFD + LI). Our results showed that the HFD-LI improved atherosclerosis by decreasing en face lesions of the aorta and reducing the accumulation of macrophages and disordered smooth muscle cells. HFD-LI also decreased iron levels, serum hepcidin levels and the serum concentration of low-density lipoprotein cholesterol (LDL-C). The use of the isobaric tag for absolute quantification (iTRAQ) proteomic method and subsequent multi-technique molecular validation indicated that many of the proteins involved in atherosclerotic inflammation, vascular remodeling, and focal adhesion had significant changes in their expression among the diet groups. Importantly, the proteins Gal-3 and VCAM1, which are key participants of atherosclerosis pathogenesis, revealed lower expression after a low-iron diet. The present findings widely support the “iron hypothesis” of atherosclerosis. Further studies are suggested to fully understand the implications of these results.

## 1. Introduction

Atherosclerosis is a chronic cardiac disease characterized by the hardening of arteries due to lipoprotein-associated plaque aggregation. Considered a first-world health concern until the recent past, atherosclerosis has now spread globally as part of an “epidemiological transition” [1]. Better hygienic conditions, vaccination, and effective management of acute infections have lowered the prevalence of contagious diseases in emerging countries; therefore, presently more people survive to endure chronic conditions, among which is atherosclerosis. In particular, progressive economic conditions in Asia, Central and South America have been accompanied by shifts from ancestral healthy diets and obesity and diabetes epidemics, together with lasting tobacco consumption. These public health threats potentiate the risks of atherosclerotic cardiovascular illness [2]. Moreover, the number of young women who are affected by coronary artery disease has recently escalated, while the aging of the worldwide population entails that the very old are now a growing percentage of the cardiac patients [3,4].

Data from the 2020 Global Burden of Disease compilation revealed that the world prevalence of ischemic cardiac illness has risen from nearly 100 million in 1990 to more than 180 million patients in 2019 [5]. However, atherosclerosis is a challenging disease with complex pathophysiology that entails inflammation, plaque formation, lipoprotein oxidation, and calcification [6], and its precise molecular mechanisms remain to be elucidated. It is now responsible for the majority of mortality around the globe and finding effective treatments has become a critical necessity.

In 1981, the “Iron Hypothesis” introduced the possibility that increased levels of stored iron may induce cardiovascular illnesses while, on the other hand, iron deficiency could offer an atheroprotective effect [7,8]. This hypothesis encouraged more studies about the role of iron in the progression of atherosclerosis, particularly in the murine and rabbit models. In mice that suffered from iron overload disease, the injection of iron chelators prevented the progress of atherosclerosis [9]. Macrophage iron reduction through iron-deficient diets or chelators also alleviated atherosclerosis [10,11]. Moreover, higher doses of iron in the diet-induced macrophage inflammation aggravated an atherosclerotic condition [12]. In rabbits, intramuscular injections of iron dextrane exacerbated the appearance of atherosclerotic lesions when in hypercholesterolemia [13], while DFO-driven iron chelation diminished the iron content of the lesions and even inhibit their development after a cholesterol diet [14]. However, there have been also conflicting results such as a high-iron diet reducing atherogenesis [15] or iron charging of macrophages not affecting this disease [16] in mice. These inconsistencies could be related to deficiencies in research modeling methods. However, existing research mainly focuses on a certain protein or mechanism, which not only invalidates the comparison of results in many cases but is also not comprehensive enough. This picture denotes the need for a refinement of the iron hypothesis and deeper molecular studies.

In the present work, we used the modeling method of dietary low iron, which is more in line with the daily human diet than the injection of iron chelators. Meanwhile, proteomic technology was used to report for the first time the overall changes in aortic tissue proteins under the condition of dietary low iron. Specifically, a quantitative proteomic approach was used to compare ApoE KO mice fed with a normal diet (ND), high-fat diet (HFD), or high-fat +low iron diet (HFD + LI), respectively.

Our objective was to reveal the underlying molecular mechanisms behind the low-iron diet and atherosclerosis alleviation. For this purpose, aortic tissues were screened to assess differentially expressed proteins between HFD/ND and HFD + LI/HFD groups. Notably, these experiments revealed that dietary low iron alleviates atherosclerosis in ApoE KO mice by down-regulating proteins involved in aortic inflammation, vascular remodeling, and focal adhesion.

## 2. Results

### 2.1. Mouse Body Weight

Mouse body weight evolution was analyzed to assess the effect of dietary iron restriction (Supplementary Figure S1) in each diet group for all 16 weeks. Compared to ND, both HFD and HFD + LI were higher than ND (*p* < 0.05, vs. ND). Food intake and energy intake were also evaluated. Food intake revealed no significant differences among the groups. The energy intake of HFD was significantly higher than ND (*p* < 0.05, vs. ND).

### 2.2. Low-Iron Diet Improves Atherosclerosis in ApoE KO Mice 

**Figure 1:**
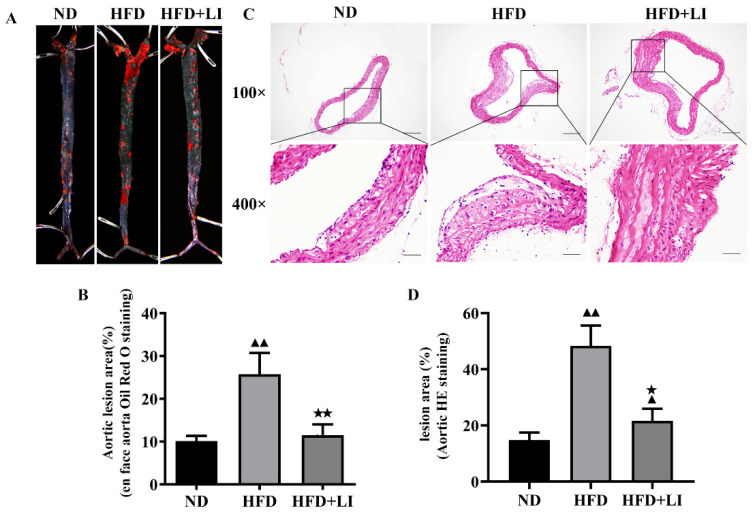
Atherosclerotic lesions in ApoE KO mice according to each diet group. (**A**) Representative images of en face oil red O staining of murine whole aortas. (**B**) Quantitative analysis of A regarding aortic lesion area (%). (**C**) Representative images of H&E staining of murine aortic atherosclerotic plaques at 100× and 400× magnification. (**D**) Relative quantification of (**B**) ^▲▲^ *p* < 0.01 vs. ND; ^★^ *p* < 0.05 vs. HFD; ^★★^ *p* < 0.01 vs. HFD. Scale bar for 100× = 200 μm; scale bar for 400× = 50 μm; n = 3. ND: normal diet, HFD: high-fat diet, HFD + LI: high-fat diet without iron supplementation.

Oil Red O staining revealed that the en face lesions of the entire aorta caused by HFD were decreased after a low iron diet (HFD + LI) (Figure 1A). Quantitatively, there was a reduction in the lesion area of more than 10% after HFD + LI when compared to HFD (*p* < 0.01), while HFD produced around 15% more lesion area than ND (*p* < 0.01) (Figure 1B). For its part, H&E staining of the aortic cross-sections showed that the HFD group had the largest plaque coverage, followed by milder plaque formation in the HFD + LI group, and with ND in the third place with the least amount of plaque (Figure 1C). According to this staining and in terms of lesion area relative to ND, there was also a significant decrease after HFD + LI versus HFD (Figure 1D).

### 2.3. Dietary Low Iron Reduces the Macrophage Accumulation versus the HFD Diet in ApoE KO Mice 

**Figure 2:**
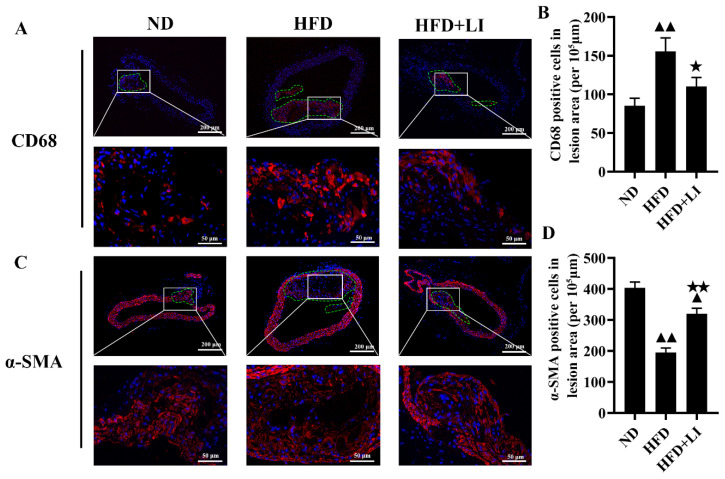
Effects of low dietary iron on ApoE KO mice aortic macrophages and smooth muscle cells. (**A**) Representative immunofluorescent staining images of macrophages in a murine atherosclerotic plaque. Nuclei were stained with DAPI (blue), and CD68-positive macrophages were revealed by Alexa Fluor 594 (red). (**B**) Quantification of (**A**) (% positive area). (**C**) Representative immunofluorescent staining images of smooth muscle cells in murine atherosclerotic plaque. Nuclei were stained with DAPI (blue), and αSMA-positive smooth muscle cells were revealed by Alexa Fluor 594 (red). (**D**): Quantification of (**C**) (percentage positive area). Scale bar = 50 μm. ^▲▲^ *p* < 0.01 vs. ND; ^★^ *p* < 0.05 vs. HFD; ^★★^ *p* < 0.01 vs. HFD. ND: normal diet-fed ApoE KO mice, HFD: high fat diet-fed ApoE KO mice, HFD + LI: high fat diet without iron supplementation-fed ApoE KO mice.ND: normal diet, HFD: high-fat diet, HFD + LI: high-fat diet without iron supplementation.

Immunofluorescence staining in murine atherosclerotic plaques was performed with CD68 and αSMA, together with DAPI. CD68 exposed a higher number of macrophages (Figure 2A) in the HFD group versus HFD + LI or ND. αSMA staining revealed that smooth muscle cells were arranged in disorder and a reduced number in the HFD group versus HFD + LI or ND (Figure 2C). Semi-quantitative results of the fluorescent staining corroborated the latter results for both antibodies (Figure 2B,D).

**Figure 3:**
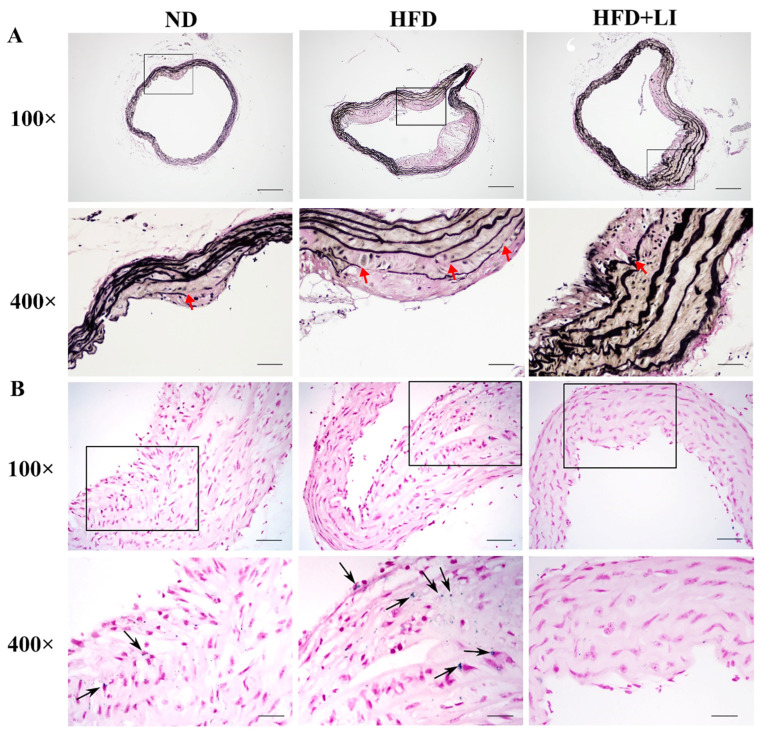
Characterization of aortic elastic fibers and iron deposition in ApoE KO mice according to each diet group. (**A**) Voerhoff von Gieson staining of mice aortic tissues. (**B**) Prussian blue staining of murine aortic tissue sections assessed iron deposition in atherosclerotic plaques (n = 4). Arrowheads indicate positive staining areas. Images are shown at 100× and 400× magnification. Scale bar for 100× = 200 μm; scale bar for 400× =50 μm. ND: normal diet, HFD: high-fat diet, HFD + LI: high-fat diet without iron supplementation.

Additionally, by comparing Figure 2A,C it can be seen that the expression level of α-SMA was significantly decreased and the expression level of CD68 was significantly increased in the atherosclerotic lesions of the HFD group (*p* < 0.01). Therefore, HFD + LI could increase α-SMA expression and decrease CD68 expression (*p* < 0.05). The above results suggested that HFD + LI could improve the stability of the plaque. On the other hand, VVG staining is an index to evaluate the structural integrity of vascular elastic fibers, and the degree of atherosclerotic plaque damage. After VVG staining, it was visible that the HFD group had the largest vascular damage, followed by HFD + LI with milder lesions, and ND with the least injury (Figure 3A).

### 2.4. Iron Parameters and Lipid Profiles 

**Figure 4:**
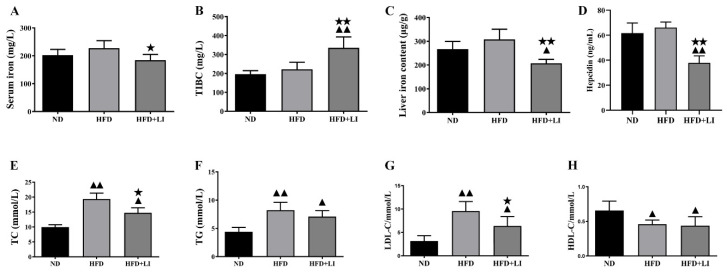
Iron parameters and lipid profiles in HFD + LI-fed ApoE KO mice. (**A**–**D**) Serum iron, serum total iron-binding capacity (TIBC), liver iron content and serum hepcidin were measured in each group (n = 6), and presented as the mean ± SD. Samples were pooled from three independent experiments. (**E**–**H**) Serum TC, TG, LDL-C, and HDL-C levels were determined in each group (n = 6), and presented as the mean ± SD. Samples were pooled from three independent experiments. ^▲^ *p* < 0.05 vs. ND; ^▲▲^ *p* < 0.01 vs. ND; ^★^ *p* < 0.05 vs. HFD; ^★★^ *p* < 0.01 vs. HFD. ND: normal diet-fed ApoE KO mice, HFD: high fat diet-fed ApoE KO mice, HFD + LI: high fat diet without iron supplementation-fed ApoE KO mice.

After Prussian blue staining of aortic tissue sections, iron deposition on atherosclerotic plaques was highest in the HFD group, followed by the ND group. No iron deposition was observed in the HFD + LI sample (Figure 3B). Meanwhile, the HFD was associated with higher serum iron when compared to the ND or the HFD + LI (*p* < 0.05) (Figure 4A). TIBC had the lowest value in the ND group, with a higher number observed in HFD (*p* < 0.01 versus ND), and an even greater one found in HFD + LI (*p* < 0.01 versus HFD) (Figure 4B). Total liver iron content in the HFD + LI group was decreased 32.5% and 41.4% compared to the ND or HFD group, respectively (Figure 4C). Hepcidin levels in HFD + LI were significantly lower than in the HFD and ND groups (*p* < 0.01) (Figure 4D).

The lipid profile analysis revealed that, when compared to a ND, HFD had nearly double the values of TC (*p* < 0.01) (Figure 4E), and also rises in TG (*p* < 0.01) (Figure 4F). In particular, TC and TG levels were significantly lower in the HFD + LI group than in the HFD group. Regarding LDL, both HFD and HFD-LI showed greater values than ND, with almost a three-time increment for HFD (*p* < 0.01) and nearly double values for HFD + LI (*p* < 0.05). However, the LDL value for HFD-LI was lower than for HFD (*p* < 0.05) (Figure 4G). In the HDL panel, results showed that both HFD and HFD-LI had lower levels than ND (*p* < 0.05) (Figure 4H).

### 2.5. iTRAQ Analysis Identified Differentially Expressed Aortic Proteins after ND, HFD, and HFD-LI

**Figure 5:**
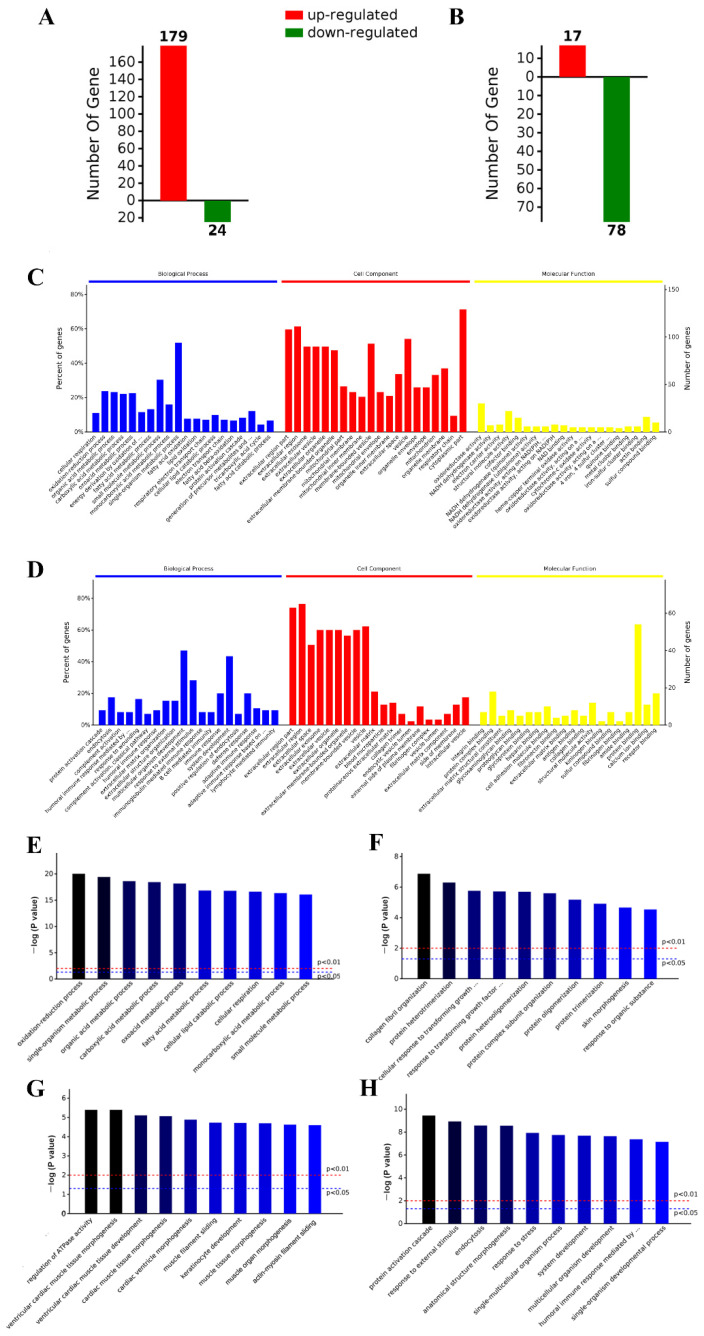
Aortic differentially expressed proteins were identified by the iTRAQ approach. HFD up- or down-regulated proteins (versus ND) (**A**). HFD-LI up- or down-regulated proteins (versus HDF) (**B**). GO enrichment analyses of the aortic differentially expressed proteins of HFD group versus ND group (**C**). GO enrichment analyses of the aortic differentially expressed proteins of HFD + LI group versus HFD group (**D**). GO enrichment analysis revealed the most relevant biological process (BP) terms of proteins up-regulated by HFD (versus ND) (**E**), proteins down-regulated by HFD (versus ND) (**F**), proteins up-regulated by HFD-LI (versus HFD) (**G**), and proteins down-regulated by HFD + LI (versus HFD) (**H**). Top 10 GO clusters (*p* < 0.05). GO, Gene Ontology.

Analysis showed significant differential expression results between the groups. The HFD cluster presented 179 up-regulated proteins and 24 down-regulated proteins versus the ND cluster (Figure 5A). Different distribution was found when HFD and HFD-LI were compared, as HFD-LI had 17 up-regulated proteins and 78 down-regulated proteins versus HFD (Figure 5B). Protein identification and further expression details can be found in Supplementary Table S1. 

### 2.6. GO and KEGG Analysis

GO enrichment analysis revealed the most relevant terms for the differentially expressed proteins regarding biological processes (BP), cellular component (CC), and molecular function (MF). This analysis was conducted for HFD versus ND (Figure 5C), and HFD + LI versus HFD (Figure 5D). In HFD versus ND, the most frequent terms were related to fatty acid metabolism (BP), cytoplasm and extracellular regions (CC), and oxidoreductase activity (MF). In HFD + LI versus HFD, the most recurrent terms were cellular development and defense (BP), extracellular space and vesicles (CC), and protein binding (MF). 

Then, a deeper iteration was performed to learn the main GO terms among the diet groups, with a special focus on the BP (Figure 5E,F). CC and MF analysis were also run (Supplementary Figure S2). Figure 5E,F show the results of HFD versus ND for differential up- and down-regulation, respectively. Figure 5G,H show the differentially up- and down-regulated proteins, respectively, for HFD + LI versus HFD. Special attention was paid to the GO terms of the proteins whose expression was increased by HFD (Figure 5E) and decreased by HFD-LI (Figure 5H). The HFD effect was mainly involved in oxidation-reduction and fatty acid metabolic processes, while the HFD-LI effect was related to external stimulus terms, including stress and immunological and also cellular organism development. 

**Figure 6:**
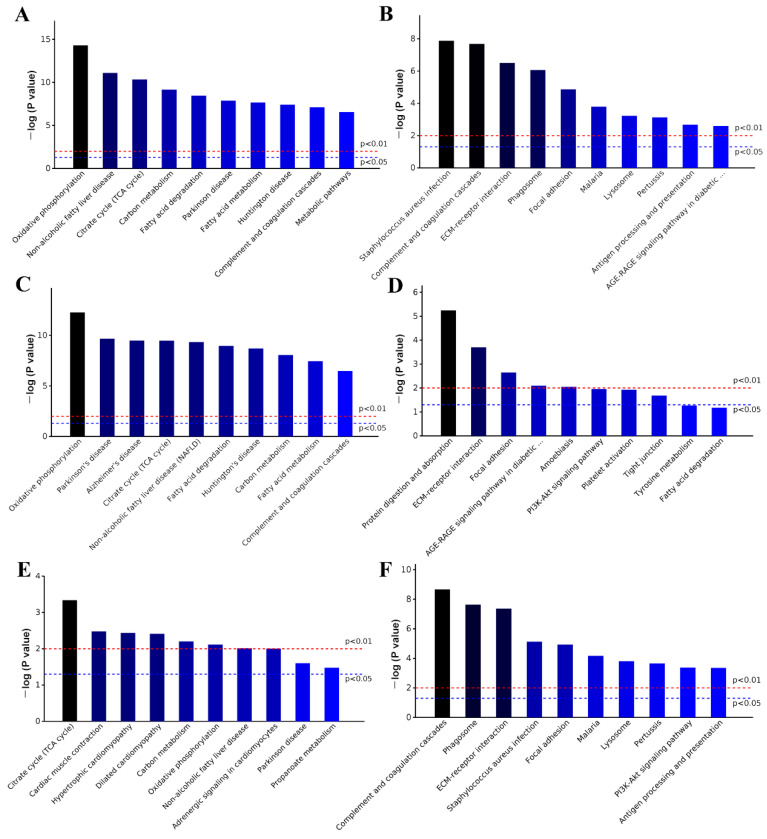
KEGG enrichment analysis of all differentially expressed proteins. KEGG enrichment analyses of the aortic differentially expressed proteins of HFD group versus ND group (**A**). KEGG enrichment analyses of the aortic differentially expressed proteins of HFD + LI group versus HFD group (**B**). KEGG enrichment results of the aortic HFD up- or down-regulated proteins (versus ND) ((**C**,**D**), respectively), and HFD-LI up- or down-regulated proteins (versus HFD) ((**E**,**F**), respectively). Top 10 clusters (*p* < 0.05). KEGG, Kyoto Encyclopedia of Genes and Genomes.

KEGG enrichment analyses of all differentially expressed proteins were performed by comparing HFD versus ND, and HFD + LI versus ND. In the first case (HFD effect), the most relevant entries were oxidative phosphorylation and diseases such as Parkinson’s and Alzheimer’s (Figure 6A). In the second case (HFD + LI effect), the main terms were *Staphylococcus aurous*, complement and regulation cascades, and others related to immunological defense (Figure 6B). In a deeper analysis, a new KEGG enrichment iteration was conducted to assess HFD versus ND, and HFD + LI versus HFD for the differential up- and down-regulation of proteins, respectively (Figure 6C–F). Special focus was paid in terms of the proteins whose expression was increased by HFD (Figure 6C) and decreased by HFD-LI (Figure 6F). In the first group (HFD-caused up-regulation), the leading terms were oxidative phosphorylation, non-alcoholic fatty liver disease, fatty acid metabolism and other diseases. In the second group (HFD-LI-caused down-regulation), the principal terms were complement and coagulation cascades, phagosome, ECM–receptor interaction, and several infections and immunological processes.

Afterward, iTRAQ analysis allowed for the screening and identification of the up- or downregulated proteins (due to HFD and HFD-LI, respectively) that had specific roles in atherosclerosis, such as inflammation, vascular remodeling and focal adhesion (Table 1; the proteins are ordered according to the value of “Ave fold change” correspondent to HFD + LI versus HFD). The data of volcano plots and PCA plots analysis are shown in the Supplementary Figures S4 and S5.

### 2.7. Validation of the Differentially Expressed Proteins Identified by iTRAQ Analysis

**Figure 7:**
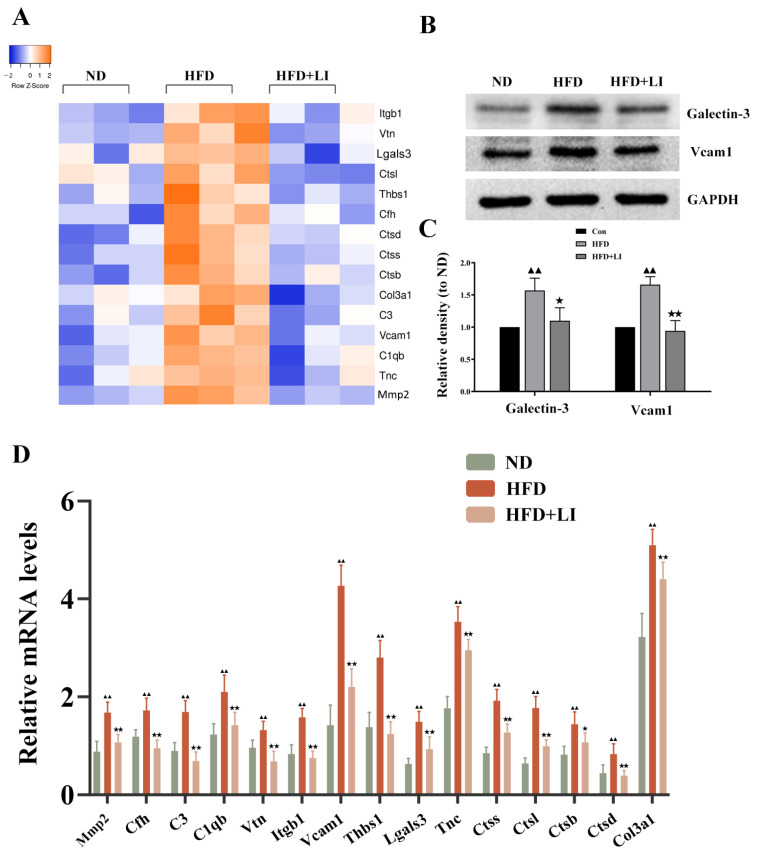
Validation of the differentially expressed proteins identified with the iTRAQ analysis and involved in aortic inflammation, vascular remodeling, and focal adhesion. (**A**) Heatmap of inflammation, vascular remodeling, and focal adhesion-related protein expression in aortic tissues in each diet group. (**B**) qRT-PCR analysis of the protein expression in aortic tissues in each diet group (n = 4). Data is presented as mean ± SD. Samples were pooled from three independent experiments. (**C**) Western blot detection of Galectin-3 (gene name: Lgals3) and VCAM1 protein expression in aortic tissue. (**D**) Quantification of protein expression presented as density relative to GAPDH.^▲▲^ *p* < 0.01 vs. ND; ^★^ *p* < 0.05 vs. HFD; ^★★^ *p* < 0.01 vs. HFD. ND: normal diet-fed ApoE KO mice, HFD: high fat diet-fed ApoE KO mice, HFD + LI: high fat diet without iron supplementation-fed ApoE KO mice.

A heatmap of the differentially expressed proteins involved in aortic inflammation, vascular remodeling, and focal adhesion was designed for each diet group (Figure 7A). The identification of these proteins by iTRAQ was validated, in principle, by qRT-PCR (Figure 7B). To validate the data from iTRAQ analysis, we randomly selected two differentially expressed proteins (Galectin-3 and VCAM1) to determine their expression levels by Western blot (Figure 7C). In accordance with iTRAQ data, it was verified that the proteins Galectin-3 and VCAM1 had greater expression in the HFD group versus the ND group (*p* < 0.01). Moreover, these proteins had lower expression in HFD + LI than in HFD (*p* < 0.05 for Galectin-3, and *p* < 0.01 for VCAM1) (Figure 7D).

Finally, a protein–protein interaction analysis was conducted on all the differentially expressed proteins between HFD and ND (Supplementary Figure S3A), and between HFD + LI and HFD (Supplementary Figure S3B). The presented diagrams show the protein-protein connections that occur with the previously disclosed KEGG pathways. 

## 3. Discussion

Since the presentation of the “iron hypothesis” almost 40 years ago, intense debate has ensued on the connection between iron and atherosclerosis. Numerous epidemiological studies have reported positive associations between the risk of developing this disease and body iron stores [17,18,19,20]. In addition, iron availability reduction by either phlebotomy, blood donation, or iron chelation has been shown to lower the risk of atherosclerosis [21,22,23]. Nevertheless, contradictory evidence has also been collected, refuting the latter associations [24] or reporting inverse findings [25]. In the case of animal research, the information is still scarce and frequently inconsistent, possibly due to variances in iron loading and the duration of the procedures among research strategies [9,12,13,16,26]. Importantly, these discrepancies are accompanied by a lack of understanding of the underlying molecular mechanisms. Here, we implemented a proteomic approach to further assess the molecular mechanisms through which a low-iron diet could alleviate atherosclerotic cardiac disease. For this purpose, ApoE KO mice fed with normal (ND), high-fat (HFD), or high-fat +low iron (HFD + LI) diets were compared. 

In our study, oil red O staining revealed that the low-iron diet improved atherosclerosis by decreasing en face lesions of the entire aorta caused by an HFD. Moreover, H&E staining of the aortic cross-sections showed less plaque formation in the HFD + LI versus HFD. This was consistent with other findings that proved that iron depletion could effectively diminish vascular degeneration and inflammation in atherosclerotic ApoE KO mice [9,16], and considerably reduce vascular lesions in atherosclerotic rabbits by lowering plasma cholesterol levels [27]. Furthermore, our immunofluorescence staining in atherosclerotic plaques showed that HFD + LI not only helped to reduce the number of macrophages but also prevented disorders and the proliferation of smooth muscle cells. The reason behind this phenomenon could be our previous finding that iron retention in plaques exacerbates atherosclerosis by triggering macrophage foaming [28], which would be prevented by a low-iron diet (HFD + LI). In line with this, no iron deposition on atherosclerotic plaques was observed in the HFD + LI sample, while iron deposition was substantial in the HFD group. Iron parameters throughout the different diets (ND, HFD, and HFD + LI) revealed that HFD had the highest serum iron levels and that these levels decreased in the HFD + LI group. Moreover, HFD + LI showed the highest TIBC values. Several previous studies have shown that during the development of atherosclerosis, hepcidin regulates the reduction of serum iron content [29]. The present study showed that serum hepcidin levels in HFD + LI were significantly lower than HFD and ND. On the other hand, no particular effect on body weight, food or energy intake was found for HFD + LI versus HFD (Supplemental Figure S1). 

Since iron has long been associated with hepatic lipid metabolic disorders [30,31,32] we assessed the lipid profiles after the different diets (ND, HFD, and HFD + LI). TC levels and LDL-C levels were significantly lower in the HFD-LI group than in the HFD group. On the other hand, there were no significant differences in the TG and HDL measurements between the HFD and HFD-LI groups. In a previous article, TC, TG, and LDL were shown to be significantly decreased in an iron-supplemented diet [33]. The potential implications of these results remain to be elucidated; however, the decreased TC and LDL indicated an anti-atherosclerosis effect of HFD-LI in ApoE KO mice. 

Regarding the proteomic analysis, the inverse proportion of up- and down-regulated proteins in the HFD and HFD + LI groups, respectively, exposed the impact of a low-iron dose in a diet in the context of atherosclerosis. As expected, the GO terms of the proteins whose expression was increased by HFD were linked to atherosclerosis development, including fatty acid metabolism and oxidoreductase activity. On the other hand, the proteins whose expression was decreased by HFD-LI were involved in responding to external stimuli, including stress and immunological events. These results in the low-iron diet group could be linked to the fact that iron inoculations in rodents frequently lower antioxidant defense mechanisms, such as catalase and SOD, in the liver and blood [34,35].

KEGG pathways data showed that the differentially expressed proteins up-regulated by HFD had expected connections with atherosclerosis development. In particular, the main KEGG term was oxidative phosphorylation, which is extensively associated with this disease and other cardiomyopathy based on human and animal research [36]. The second most relevant pathway term was “non-alcoholic fatty liver disease”, which is widely known for inducing systemic dyslipidemia and being an important risk factor for atherosclerosis [37]. For its part, the differentially expressed proteins down-regulated by HFD-LI were essentially involved in defense mechanisms, as discussed in the previous paragraph. 

The subsequent screening and identification of the proteins that were up- or downregulated by HFD and HFD-LI allowed the listing of numerous proteins involved in atherosclerotic progression through inflammation, tissue remodeling, and focal adhesion. Two proteins, Galectin-3 (Gal-3) and VCAM1, showed important mRNA expression and were further assessed. In recent years, Gal-3 has been positioned as a novel inflammatory factor that participates in intravascular inflammation, macrophage activation, lipid endocytosis, cellular proliferation, monocyte chemotaxis, and cell adhesion during atherosclerosis [5]. On the other hand, aortic VCAM1 expression has been long correlated with the severity of the atherosclerotic disease and cardiovascular risk factors; therefore, it plays a role in the pathogenesis of atherosclerosis [38]. The fact that these proteins had greater expression in the HFD group versus the ND group agrees with the latter backgrounds. Importantly, the lower expression of these proteins in the HFD + LI group (versus HFD) largely supports a dietary iron restriction alleviating atherosclerosis. 

There are some limitations in this work. First, further studies are needed to better understand the molecular mechanisms that connect low iron concentrations and atherosclerotic outcomes. Second, the present results require additional validation in cellular models. 

## 4. Materials and Methods

### 4.1. Animals

Animals were treated following the Guiding Principles in the Care and Use of Animals approved by the Animal Care and Use Committee of the Central South University (Changsha, Hunan, China) (approval #XYGW-2021-82). Six week-old female ApoE KO mice (18–20 g) with a C57BL/6J genetic background (n = 36) were obtained from Beijing Vital River Laboratory Animal Technology Co., Ltd. (Beijing, China). Sex differences have been reported in mouse atherosclerosis studies using ApoE KO mice with a C57BL/6J genetic background [39]. The most widely reported sex effect on atherosclerosis is that female ApoE KO mice develop mature fibrofatty atherosclerotic plaques as early as 16 weeks of age, and they have significantly larger lesions than their male counterparts [40,41]. Therefore, only female ApoE KO mice were used in the present study. After one week of acclimatization with a chow diet and ad libitum water, the mice were randomly divided into three groups (n = 12 per group) and fed as follows: (1) normal chow diet (ND), with an iron content of 200 mg/kg (based on the Research Diets # D12450J formulation); (2) high-fat diet (HFD), an atherogenic diet containing 21% fat and 0.15% cholesterol, and iron content of 200 mg/kg (based on the Research Diets #D12079B formulation); or (3) high-fat diet without iron supplementation (HFD + LI), with an iron content of <20 mg/kg (modified the Research Diets #D12079B formulation without iron supplementation). The feed was provided by Beijing Keao Xieli Feed Co., Ltd. (Beijing, China). After one week of adaptive feeding, all mice were continuously fed with ND, HFD, or HFD + LI for 16 weeks. All animals were maintained under a specific pathogen-free environment, standard light/darkness cycle (12 h/12 h), and free access to food and water.

### 4.2. Atherosclerosis Lesion Evaluation 

After 16 weeks, four mice were randomly selected from each group for en face aorta oil red O staining. Following a previous procedure [42], the heart and aorta were extracted after perfusion through the left ventricle with phosphate-buffered saline (PBS) and 4% paraformaldehyde. Afterward, whole aortas were separated from the heart, opened longitudinally, and treated with oil red O staining to undergo an en face morphometric analysis to evaluate atherosclerotic lesions. Images were captured with a Canon IXUS 220 HS camera, and the lesion size of the aorta was analyzed and quantified by Image-Pro Plus 6.0.5 (Media Cybernetics, Inc., Rockville, MD; RRID:SCR-007369).

### 4.3. Hematoxylin-Eosin and Immunofluorescence Staining

Aortic lesion area and macrophage/smooth muscle cells quantitation were determined by hematoxylin and eosin (H&E) staining and immunofluorescence staining, respectively, as described previously [42]. Briefly, samples were obtained from the aortic arch tissue, fixed in 4% paraformaldehyde, inserted in tissue freezing medium optimum cutting temperature compound (OCT), and finally segmented into consecutive 5 μm-thick pieces at 20 °C. Every six sections were reserved for H&E staining or immunofluorescence staining and observed under a microscope (Olympus, Tokyo, Japan). The antibodies used in this study were α-SMA (1:250, ab124964, Abcam) and CD68 (1:100, ab283654, Abcam) to color smooth muscle cells and macrophages, respectively, and revealed by Alexa Fluor 594 (red). Furthermore, 0.1 mg/mL diamidino-2-phenylindole (DAPI) in PBS solution was employed to mark cell nuclei, as described previously [42]. Quantitative measurements were randomly conducted on six sections using Image-Pro Plus 6.0.5 (Media Cybernetics, Inc., Rockville, MD, USA; RRID:SCR-00736).

### 4.4. Lipid Measurement

Blood samples were clotted on ice for 15 min and centrifuged at 200× *g* for 10 min at 4 °C. Then, the supernatant was collected to measure serum total cholesterol (TC), triglyceride (TG), low-density lipoprotein cholesterol (LDL-C), and high-density lipoprotein cholesterol (HDL-C). Quantification was performed with commercial kits following the manufacturer’s instructions.

### 4.5. Quantification of Serum Iron, Liver Iron Content, Total Iron-Binding Capacity (TIBC), and Iron Deposition in Atherosclerotic Plaques

Serum iron and total iron-binding capacity (TIBC) were detected by iron/TIBC assay kits following the manufacturer’s instructions. Total liver iron content was measured through flame atomic absorption spectrometry with an iron hollow cathode lamp. To assess iron deposition in atherosclerotic plaques, Prussian blue staining was performed on aortic tissue sections. For this purpose, aortas were excised, embedded in OCT compound, frozen on dry ice, and finally kept at −80 °C. Finally, aortic tissues were segmented into adjacent sections (5-μm) and stained.

### 4.6. Verhoeff’s Van Gieson Staining

Verhoeff’s van Gieson (VVG) staining allows evaluating the structural integrity of vascular elastic fibers, and the levels of atherosclerotic plaque damage. Aortic arch tissue sections were stained with Verhoeff’s Van Gieson (VVG) solution (saturated picric acid, 1% acid fuchsine, and distilled water) as previously described [43]. Briefly, after 30 min of staining with VVG, the samples were differentiated with 2% ferric chloride for 1 min and gently rinsed with the tap running water. To monitor the degree of dyeing, color changes were analyzed under a microscope. Then, VVG was successively applied for 3 min of staining. Finally, the samples were dehydrated in xylene and graded alcohols.

### 4.7. Protein Digestion and iTRAQ Labeling 

Aortic tissues (n = 3) were obtained from each group for proteomics analysis. Aortic tissues were ground to powder in a mortar using lipid nitrogen. Then, 150 mg of powder from each sample was combined with lysis buffer (7 M urea, 2 M thiourea), 4% (*w*/*v*) CHAPS, 40 mM Tris base, and 40 mM dithiothreitol (DTT), to be subsequently sonicated 10× for 5 s with a 10 s stop in an ice-water bath, and centrifuged at 14,000 rpm, 4 °C for 60 min. The supernatant was reserved and stored at −80 °C until use. Protein concentrations were calculated and optimized by the Bradford assay. A supernatant volume from each sample that comprised 100 μg of protein was digested overnight with trypsin at 1:50. Tryptic peptides were vacuum-dried and labeled using 8-plex iTRAQ chemicals (AB SCIEX, Framingham, MA, USA) under the manufacturer’s instructions. Afterward, 70 mL of isopropanol was employed to solubilize isobaric tags. Peptides from the ND, HFD, and HFD + LI groups, respectively, were labeled with 113, 114 and 115 iTRAQ tags, incubated at room temperature for 2 h, mixed at equivalent ratios, and dried by vacuum centrifugation [33].

### 4.8. LC-MS/MS Analysis

An Easy Nano Liquid Chromatography apparatus (nLC) combined with a Q-Exactive mass spectrometer (Thermo Fisher Scientific, San Jose, CA, USA) was utilized for the LC-MS/MS analysis. A peptide mixture containing 5 μg was injected into a C18-reversed-phase column (Thermo Scientific Easy Column; 75 μm inner diameter, 3 μm resin, 15 cm long) in buffer A (0.1% formic acid) and split with a linear gradient of buffer B (84% *v*/*v* acetonitrile; 0.1% *v*/*v* formic acid). The flow rate was 300 nL/min. Gradients were established as mobile phase B at 0–35% from 0 to 50 min, mobile phase B at 35–100% from 50 to 55 min, and finally mobile phase B at 100% from 55 to 60 min. Mass spectra were obtained in 60 min in a data-dependent mode. The automatic gain control (AGC) objective was set as 1 × 10^6^, and the highest injection time was established as 50 ms. The full scan was surveyed using a mass-to-charge ratio range of 400 to 1500 *m*/*z* and a mass resolution of 70,000 at 200 *m*/*z*. The MS/MS scans were performed with a resolution of 17,500 at 200 *m*/*z*, a 2 *m*/*z* isolation window, and a 30 eV normalized collision energy [33].

### 4.9. Protein Identification and Quantification 

Raw MS files were analyzed using the Xcalibur 2.2 SPI system. The MS/MS spectra were searched against UniProt database (http://www.uniprot.org/uniprot/, accessed on 14 November 2019) using Proteome Discoverer software (Version PD1.4, Thermo Scientific, Waltham, MA, USA). The relative quantification analysis was performed on the peak areas of the reporter ions to acquire the total proteins of each group with a relative ratio. A protein was considered differentially expressed with a fold change of >1.2 or <0.83 when the condition of at least two unique peptides was satisfied and the *p*-value was < 0.05.

### 4.10. Gene Ontology (GO) and Kyoto Encyclopedia of Genes and Genomes (KEGG) Pathway Analysis

GO (www.geneontology.org) (accessed on 14 November 2019) performs functional analysis through GO categories to inform the most relevant features (enrichment) of gene and gene products, including biological processes, molecular function, and cellular components. We applied GO categories to our differentially expressed proteins to analyze their potential biological roles. Moreover, we implemented the KEGG database (http://www.genome.jp/kegg/) (accessed on 14 November 2019) to organize and assemble these identified differentially expressed Proteins. GO term enrichment and KEGG analysis showed *p*-values (significant recommended *p*-value < 0.05).

### 4.11. qRT PCR 

Total RNA was extracted from aortic tissues using TRIzol reagent (Thermo Fisher) under the manufacturer’s instructions. For quantitative reverse transcription-PCR (qRT-PCR), a SYBR Green-based kit (TaKaRa BIO Inc., Dalian, China) was employed to assess messenger RNA (mRNA) expression through a 7900HT instrument (Applied Biosystems, Forster, CA, USA). The expression of each target gene was calculated by the comparative 2^−ΔΔCt^ method using β-actin as a normalizer. The sequences corresponding to the forward and reverse primers used can be found in Table 2.

### 4.12. Western Blot

Changes in the protein levels among different groups were verified by Western blot. For this purpose, total protein was extracted from aortic tissues from each diet group using a lysis buffer, and the protein concentration in the supernatant was calculated utilizing a BCA protein assay kit (Vazyme, USA). Then, 25 µg of protein from each group was separated using 10% SDS-PAGE gels, to be subsequently relocated to PVDF membranes. This was first incubated with skim milk (5%) for 1 h at room temperature, then with primary antibodies for Galectin-3 (1:5000, ab76245, Abcam) and Vcam1 (1:4000, ab134047, Abcam) at 4 °C, overnight, and finally with the correspondent secondary antibodies. Visualization was performed using an ECL detection reagent (Yeasen Biotechnology, Shanghai, China), and measurements were made by ImageJ software 1.48 (National Institutes of Health) [43].

### 4.13. Statistical Analysis

The SPSS 18.0 software was utilized for the statistical analysis and all data was stated as mean ± standard deviation of replicate experiments. The one-way analysis of variance (ANOVA) or the Student’s *t*-test were the methods to compare between groups considering continuous variables. Statistical significance was acknowledged when *p* < 0.05 [33].

## 5. Conclusions

This work broadly supports dietary iron restriction as a way to alleviate atherosclerosis through a proteomic approach. Quantitative proteomic analysis with subsequent multi-technique molecular validation revealed significant differences in the protein expression between the HFD-LI, HFD, and ND groups. In particular, many of the proteins involved in atherosclerosis progression and with roles in inflammation, vascular remodeling and focal adhesion had important changes in their expression among the diet groups. Most notably, Gal-3 and VCAM1, which have major participation in the pathogenesis of atherosclerosis, showed lower expression after a low-iron diet. Further studies are suggested to better understand these results.

## Figures and Tables

**Table 1:** iTRAQ analysis identified the differentially expressed proteins involved in aortic inflammation, vascular remodeling and focal adhesion in murine aortic tissues.

Protein Name	Gene Name	NCBI Accession	Roles in Atherosclerosis	HFD vs. ND			HFD + LI vs. HFD		
				Ave Fold Change		*p* Value	Ave Fold Change		*p* Value
Collagen alpha-1(IV)chain	Col4a1	P02463	vascular remodeling/Focal adhesion	1.27	↑	0.0122	0.83	↓	0.0039
Cathepsin S	Ctss	F6WR04	vascular remodeling	1.22	↑	0.0108	0.82	↓	0.0464
Collagen alpha-1(III)chain	Col3a1	P08121	Focal adhesion	1.22	↑	0.0160	0.82	↓	0.0099
Vitronectin	Vtn	P29788	inflammation/vascular remodeling/Focal adhesion	1.23	↑	0.0061	0.81	↓	0.0048
Complement C3	C3	P01027	inflammation/vascular remodeling	1.23	↑	0.0002	0.81	↓	0.0002
Cathepsin L1	Ctsl	P06797	vascular remodeling	1.43	↑	0.0023	0.80	↓	0.0437
Integrin beta-1	Itgb1	P09055	inflammation/vascular remodeling	1.31	↑	0.0141	0.80	↓	0.0294
Cathepsin B	Ctsb	P10605	vascular remodeling	1.39	↑	0.0012	0.80	↓	0.0007
Myosin regulatory light chain 2,atrial isoform	Myl7	Q9QVP4	Focal adhesion	1.25	↑	0.0200	0.80	↓	0.0001
Cathepsin D	Ctsd	P18242	vascular remodeling	1.47	↑	<0.0001	0.78	↓	0.0001
Complement factor H	Cfh	E9Q8I0	inflammation	1.28	↑	0.0008	0.76	↓	0.0003
Coagulation factor XIII B chain	F13b	Q07968	inflammation	1.58	↑	0.0005	0.75	↓	0.0070
Complement C1q subcomponent subunit B	C1qb	P14106	inflammation	1.35	↑	0.0009	0.71	↓	0.0018
Beta-2-microglobulin	B2m	P01887	vascular remodeling	1.32	↑	0.0267	0.69	↓	0.0117
Chondroadherin	Chad	O55226	vascular remodeling/Focal adhesion	1.70	↑	0.0003	0.69	↓	0.0007
Thrombospondin 1	Thbs1	Q80YQ1	inflammation/vascular remodeling/Focal adhesion	1.39	↑	<0.0001	0.69	↓	<0.0001
Vascular cell adhesion protein 1	Vcam1	Q544V4	inflammation/Focal adhesion	1.54	↑	0.0089	0.68	↓	0.0150
Tenascin	Tnc	Q80YX1	vascular remodeling/Focal adhesion	1.34	↑	0.0001	0.68	↓	<0.0001
Mannose-binding protein A	Mbl1	P39039	inflammation/vascular remodeling	1.21	↑	0.0044	0.67	↓	0.0036
Complement C1q subcomponent subunit A	C1qa	P98086	inflammation	1.45	↑	0.0149	0.66	↓	0.0081
Tubulin beta-3 chain	Tubb3	Q9ERD7	vascular remodeling	1.47	↑	0.0027	0.66	↓	0.0007
Galectin-3	Lgals3	P16110	vascular remodeling/Focal adhesion	1.23	↑	<0.0001	0.66	↓	<0.0001
V-type proton ATPase 16 kDa proteolipid subunit	Atp6v0c	P63082	vascular remodeling	1.65	↑	0.0241	0.65	↓	0.0320
Cartilage oligomeric matrix protein	Comp	Q9R0G6	inflammation/vascular remodeling/Focal adhesion	1.69	↑	0.0013	0.58	↓	0.0018
72 kDa type IV collagenase	Mmp2	P33434	inflammation/vascular remodeling	1.42	↑	<0.0001	0.58	↓	0.0018

Note: Arrow “↑” represents upregulated proteins while Arrow “↓” represents downregulated proteins.

**Table 2:** Real-time quantitative PCR primer sequences.

Gene	Forward Primer 5′-3′	Reverse Primer 5′-3′
Mmp2	GTCGCCCCTAAAACAGACAA	GGTCTCGATGGTGTTCTGGT
Cfh	CGTGAATGTGGTGCAGATGGG	AGAATTTCCACACATCGTGGCT
C3	AGCAGGTCATCAAGTCAGGC	GATGTAGCTGGTGTTGGGCT
C1qb	ATAAAGGGGGAGAAAGGGCT	CGTTGCGTGGCTCATAGTT
Vtn	TGCAAGCCCCAAGTAACGCG	TGCCGTCCGTCCGAGGATTT
Itgb1	TGCTTAGTCTTACTGACAGAGG	CAATCAGCGATCCACAAACC
Vcam1	TTGGGAGCCTCAACGGTACT	GCAATCGTTTTGTATTCAGGGGA
Thbs1	GGGGAGATAACGGTGTGTTTG	CGGGGATCAGGTTGGCATT
Lgals3	GATCACAATCATGGGCACAG	ATTGAAGCGGGGGTTAAAGT
Tnc	TCATTGTGGGTCCAGATACC	GGAGTCCAATTGTGGTGAAG
Ctss	ATAAGATGGCTGTTTTGGATG	TTCTTTTCCCAGATGAGACGC
Ctsl	GTCAACATATTGGTCAAGCCGC	CCACTCCAATCCCAAGTAAGGC
Ctsb	TGCCGAGAGTGGAACACAC	AAAAGCAGCTGGACCCTACA
Ctsd	AGAGCCAGGAGAACTTTGAGCC	GAACTCATTGCGGACCACTTTG
Col3a1	GAGCTCGGTGTAAACTTTCCCTA	CTTAAAGAATCGCTTGGCCTCA
β-actin	TTCGTTGCCGGTCCACACCC	GCTTTGCACATGCCGGAGCC

## Data Availability

The datasets used and/or analysed during the current study are available from the corresponding author on reasonable request.

## Supplementary Materials

The following supporting information can be downloaded at: https://www.mdpi.com/article/10.3390/ijms232415915/s1.
