# Supplementary material for: Iron Restriction Alleviates Atherosclerosis in ApoE KO Mice: An iTRAQ Proteomic Analysis
Source: Int J Mol Sci. 2022 Dec 14;23(24):15915. doi: 10.3390/ijms232415915 (PMC9786058; doi:10.3390/ijms232415915)
Supplement: Supplementary file 1 [file ijms-23-15915-s001.zip › Supplemental Figure legends.pdf]

Supplemental Figure legends

Supplemental Figure S1. Effects of dietary iron restriction on body weight (A), food intake (B) and energy intake (C) in ApoE KO mice. ▲*P* <0.05 HFD vs. ND; △ *P* <0.05 HFD+LI vs. ND.

Supplemental Figure S2. GO terms distribution

GO enrichment analysis revealed the most relevant cellular component (CC) terms of proteins up- or down-regulated by HFD (versus ND) (A and B, respectively), and up- or down-regulated by HFD+LI (versus HFD) (C and D, respectively). Go analysis also displayed the most representative molecular function (MF) terms of proteins up- or down-regulated by HFD (versus ND) (E and F, respectively), and up- or down-regulated by HFD-LI (versus HFD) (G and H, respectively). Top 10 gene ontology clusters ($p < 0.05$).

Supplemental Figure S3. Protein-protein interaction analysis of all aortic differentially expressed proteins.

Interaction analysis correspondent to A: HFD versus ND, and B: HFD+LI versus HFD.

Supplemental Figure S4. Volcano plots

Volcano plots correspondent to A: HFD versus ND, and B: HFD+LI versus HFD.

Supplemental Figure S5. Principal components analysis (PCA) plots

2D-PCA plots correspondent to A: HFD versus ND, and C: HFD+LI versus HFD.

3D-PCA plots correspondent to B: HFD versus ND, and D: HFD+LI versus HFD.
