# Supplementary figures and images for: Iron Restriction Alleviates Atherosclerosis in ApoE KO Mice: An iTRAQ Proteomic Analysis
Source: Int J Mol Sci. 2022 Dec 14;23(24):15915. doi: 10.3390/ijms232415915 (PMC9786058; doi:10.3390/ijms232415915)

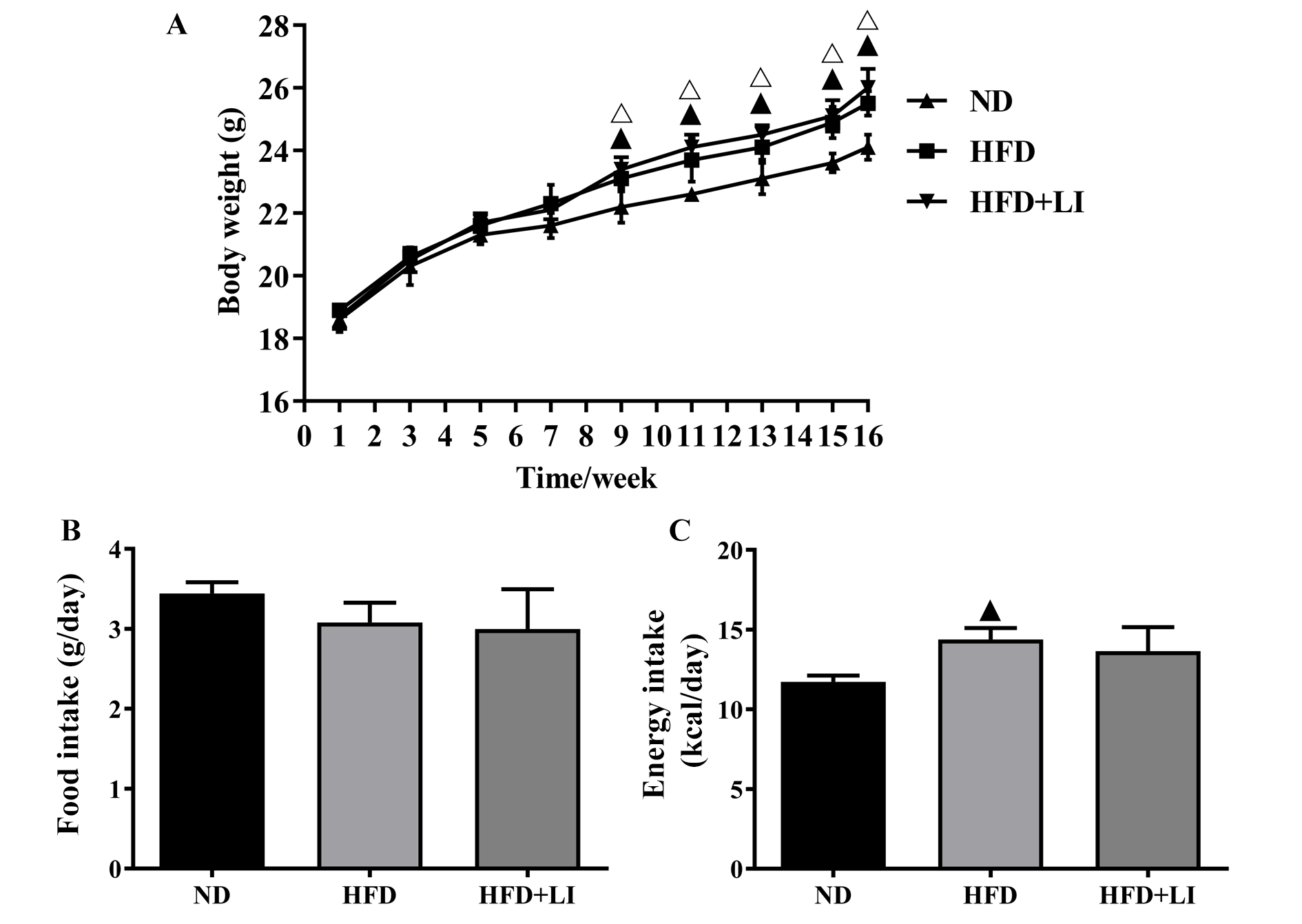

Supplement: Supplementary file 1 [file ijms-23-15915-s001.zip › Supplementary FigS1-Effects of dietary iron restriction on body weight (A), food intake (B) and energy intake (C) in ApoE KO mice..tif]

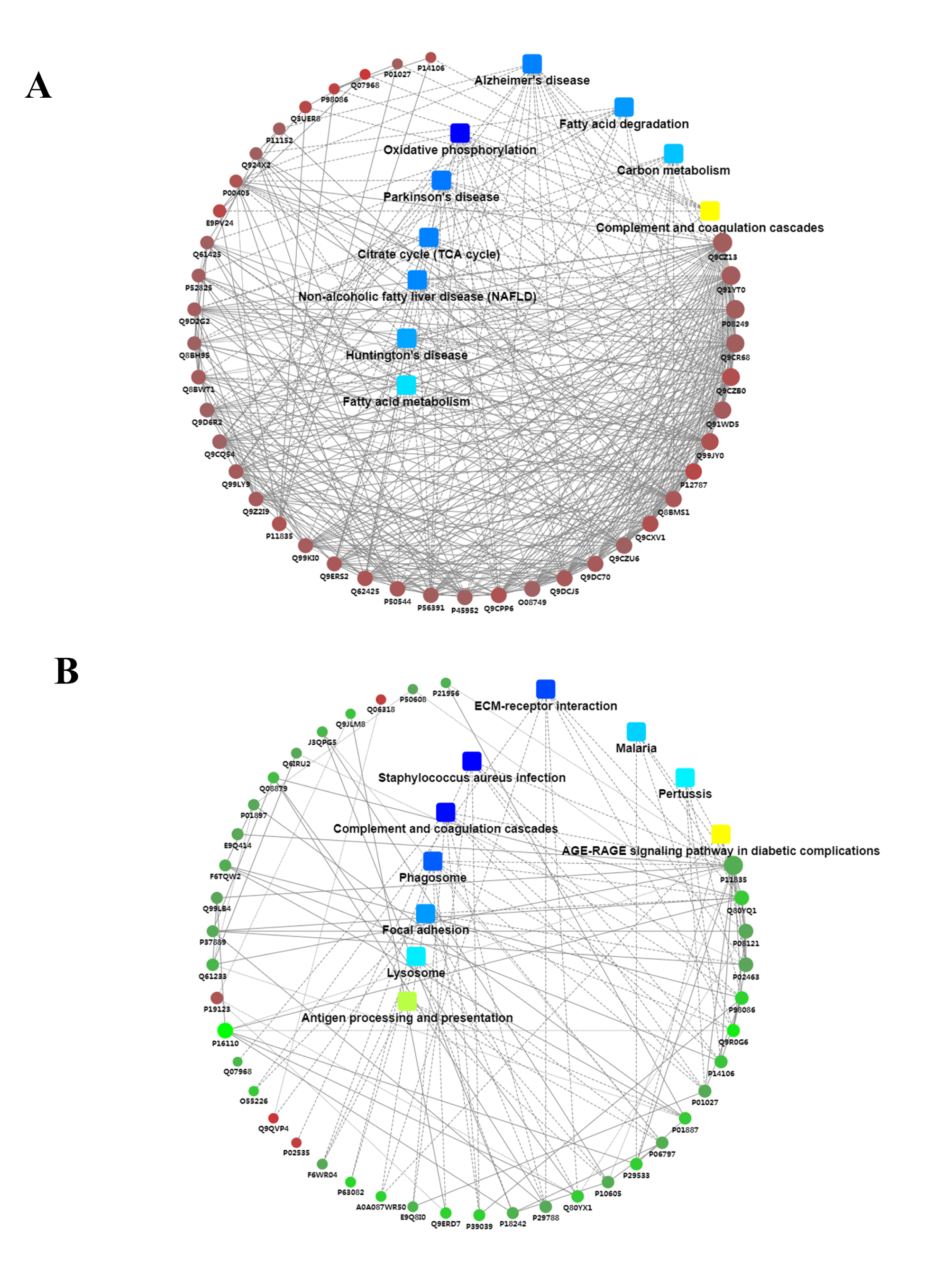

Supplement: Supplementary file 1 [file ijms-23-15915-s001.zip › Supplementary FigS3-Protein-protein interaction analysis of all aortic differentially expressed proteins.tiff]

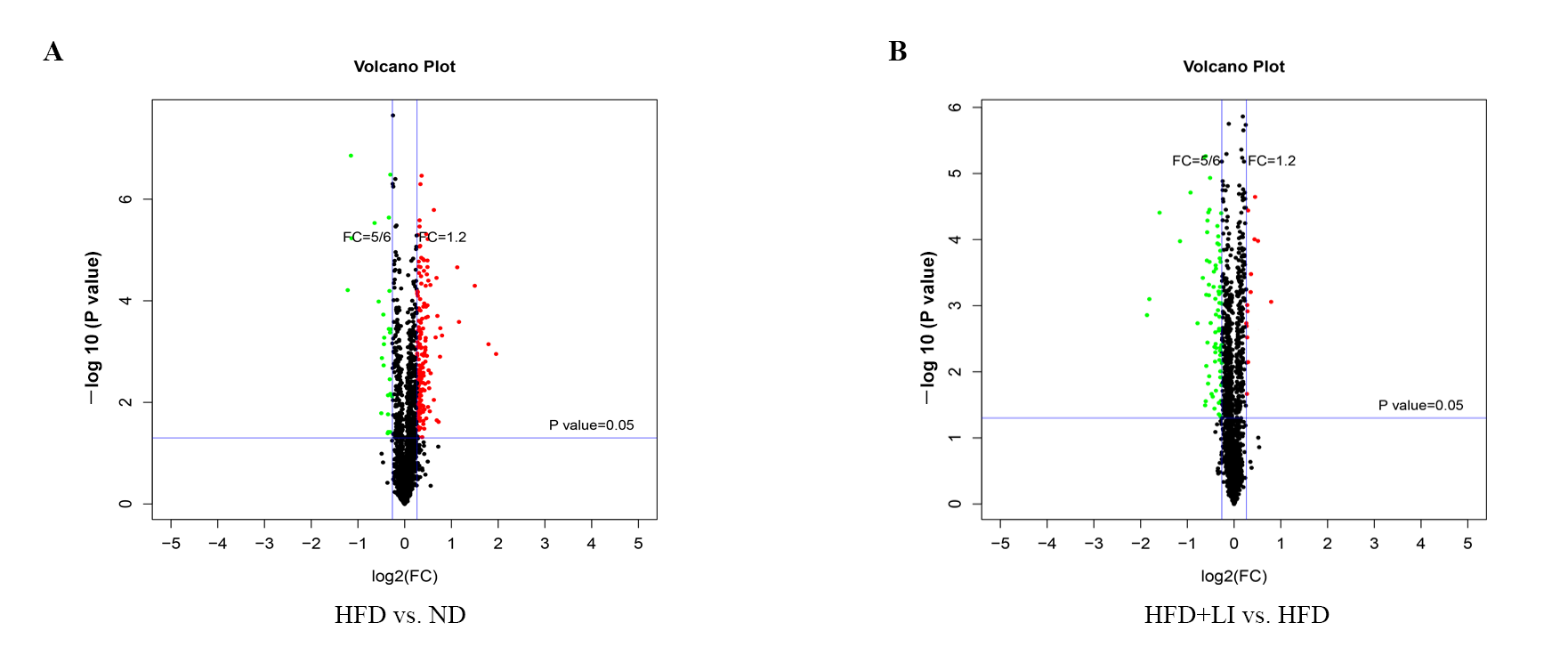

Supplement: Supplementary file 1 [file ijms-23-15915-s001.zip › Supplementary FigS4-volcano plot.tif]

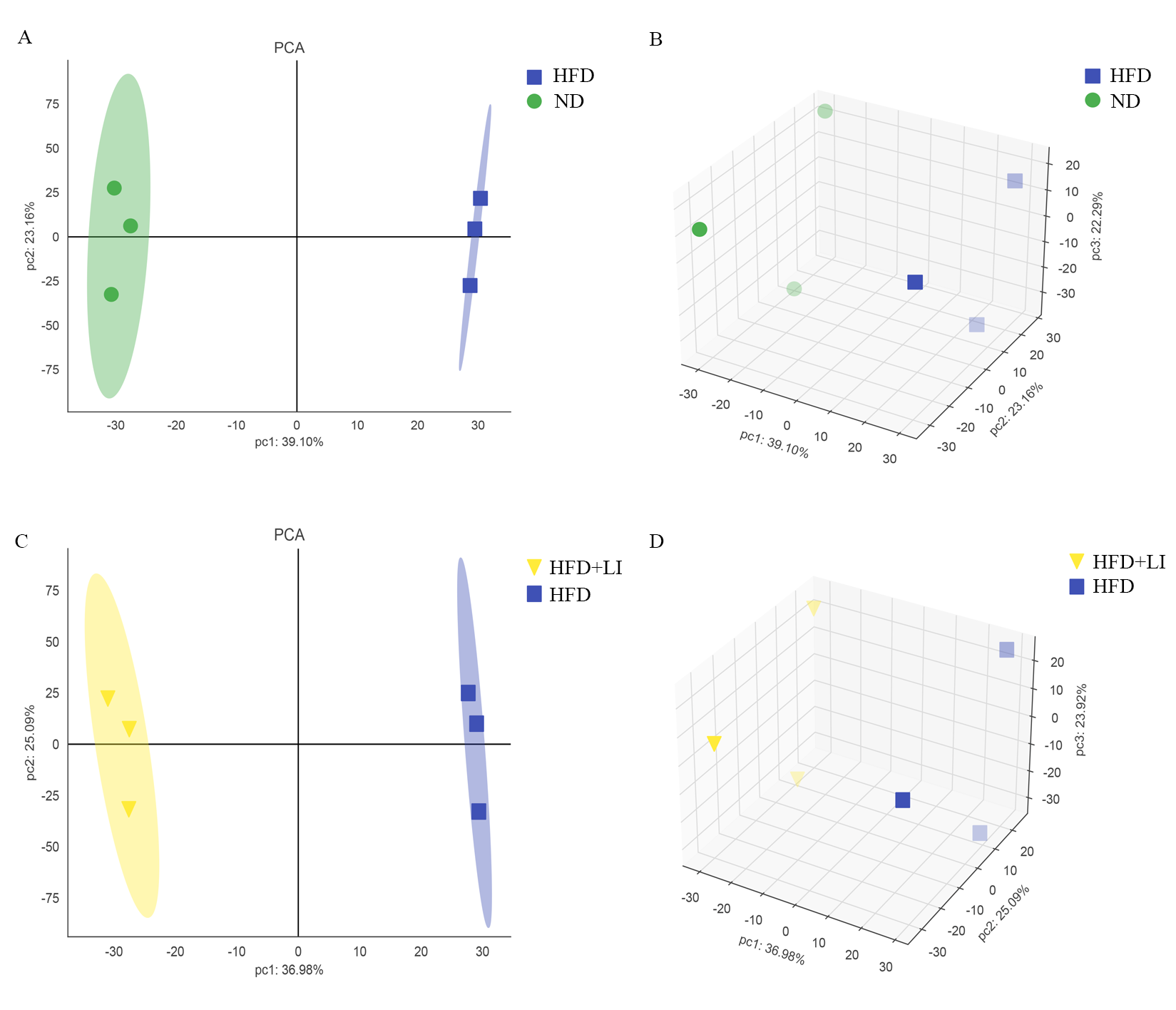

Supplement: Supplementary file 1 [file ijms-23-15915-s001.zip › Supplementary FigureS5-PCA.tif]

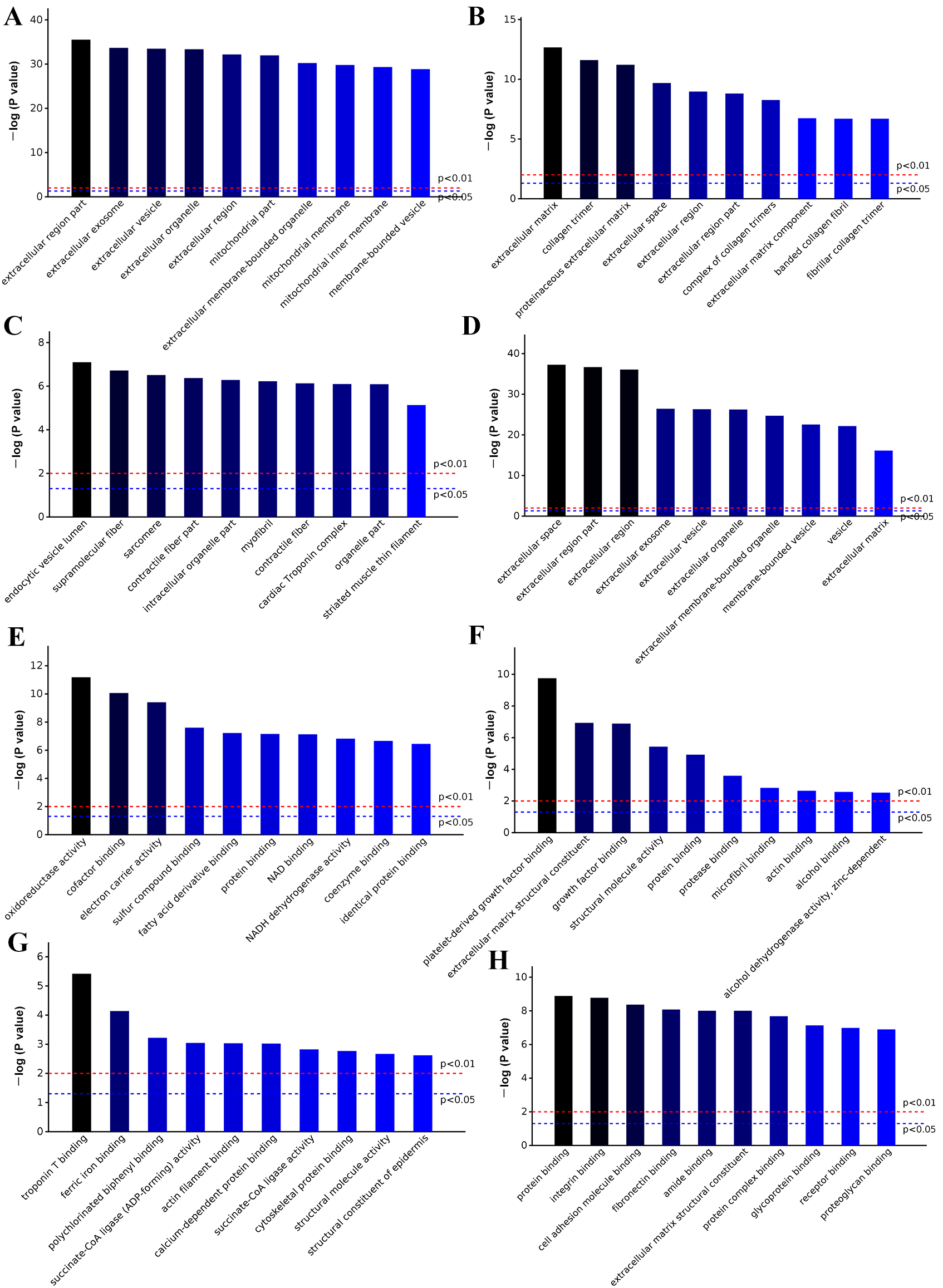

Supplement: Supplementary file 1 [file ijms-23-15915-s001.zip › SupplementaryFigS2-Go terms distribution.tif]
